# Antimicrobial Resistance among Community-Acquired Uropathogens in Mashhad, Iran

**DOI:** 10.1155/2020/3439497

**Published:** 2020-10-05

**Authors:** Mohammad Moein Vakilzadeh, Amirhossein Heidari, Ali Mehri, Matin Shirazinia, Fereshte Sheybani, Ehsan Aryan, HamidReza Naderi, Mona Najaf Najafi, Marjan Varzandeh

**Affiliations:** ^1^Faculty of Medicine, Mashhad University of Medical Sciences, Mashhad, Iran; ^2^Department of Infectious Diseases and Tropical Medicine, Faculty of Medicine, Mashhad University of Medical Sciences, Mashhad, Iran; ^3^Clinical Research Unit, Faculty of Medicine, Mashhad University of Medical Sciences, Mashhad, Iran; ^4^Antimicrobial Resistance Research Center, Faculty of Medicine, Mashhad University of Medical Sciences, Mashhad, Iran

## Abstract

**Background:**

Antimicrobial resistance among community-acquired uropathogens is an emerging concern over the past decades that warrants a continuing reevaluation of the appropriateness of recommended empiric antimicrobial regimens for treatment of urinary tract infections (UTIs).

**Aims:**

To describe the microbial spectrum and resistance profile of community-acquired uropathogens and predictors of isolation of resistant strains.

**Methods:**

Between October 2017 and June 2019, individuals who visited the outpatient clinics for diagnosis of UTIs or screening of asymptomatic bacteriuria were included in the study if they were tested for urine culture in one of the three main medical diagnostic laboratories of Mashhad, Iran. The standard disk diffusion antimicrobial susceptibility testing was used, with the Clinical and Laboratory Standards Institute (CLSI) threshold cutoffs for susceptibility of isolated uropathogens.

**Results:**

Three hundred thirty cases were included with a median age of 47 years. Two hundred seventy-six (83.6%) were female. The most common isolated uropathogens were *Escherichia coli* in 201 (60.9%) cases and *Klebsiella* species in 46 (13.9%) cases. *E. coli* isolates showed the highest rates of susceptibility to nitrofurantoin (89.3%), cefixime (75%), and gentamicin (72.4%). Exposure to antibiotics in the past 3 months was a predictor of resistance to ciprofloxacin (OR: 2.8, 95% CI: 1.33–6.28), and older age was a predictor of resistance to TMP-SMX (OR: 2.1, 95% CI: 1.07–3.97) among *E. coli* isolates. *Conclusion. E. coli* and *Klebsiella* species accounted for about two-thirds of community-acquired uropathogens. In regard to the high susceptibility rates, nitrofurantoin was identified as the first-choice agent for empiric treatment of community-acquired cystitis, while cefixime and gentamicin might be the second-choice alternatives. Ciprofloxacin and TMP-SMX, on the other hand, cannot be considered appropriate agents for empiric therapy of community-acquired UTIs, particularly in those who had exposure to antibiotics in the past 3 months and the elderly.

## 1. Introduction

Urinary tract infection (UTI) is the most common bacterial infection encountered in the ambulatory care setting [1], with a lifetime incidence of 50–60% in adult women [2]. It creates a significant social and personal burden, with a substantial number of medical visits, increased absenteeism, and negative impacts on quality of life [2]. Emergence of antimicrobial resistance among uropathogens, particularly those causing community-acquired UTIs, is one of the most important factors impacting the management of UTIs over the past decades [3].

In the appropriate clinical setting, the diagnosis of UTIs is typically made using both urinalysis and urine culture with susceptibility testing. Growth of bacteria on urine culture supports the diagnosis of UTIs, and susceptibility testing is considered essential to ensuring appropriate antimicrobial treatment [4]. However, empiric therapy is considered the standard management approach of acute community-acquired uncomplicated cystitis [1, 5, and 6]. The recommendation on management of uncomplicated cystitis is based on two important principles. First, the microbiological spectrum of acute uncomplicated UTIs is highly predictable, and second, the susceptibility pattern of these organisms is relatively predictable [7].

The appropriateness of the first-line agents for empiric therapy of UTIs is determined based on the local resistance rates of uropathogens. In this regard, thresholds have been suggested for the prevalence of resistance among uropathogens in a community above which some are not recommended; for an antibiotic to be considered a first-line empirical treatment for urinary tract infections, resistance should not exceed 10–20% in the most likely infecting strains [5]. Thus, local resistance surveillance of uropathogens and a continuing reevaluation of the appropriateness of the recommended empiric antimicrobial regimens are critical for informing empirical antimicrobial decisions [5, 8]. Unfortunately, there is no antimicrobial resistance surveillance system for monitoring of community-acquired uropathogens in Iran. This results in lack of a regional protocol for the appropriate management of community-acquired UTIs in the country. In this study, we describe the microbial spectrum and resistance profile of community-acquired uropathogens as well as predictors of isolation of resistant uropathogens in Mashhad, Iran.

## 2. Methods

This is a cross-sectional study, and sampling was carried out using the census sampling method. The study was conducted between October 2017 and June 2019 in three main public medical diagnostic laboratories of Mashhad, Iran. Mashhad is the second largest city of Iran that is located in the northeast of the country. Individuals who visited the outpatient clinics for diagnosis of symptomatic UTIs or screening of asymptomatic bacteriuria were included consecutively if their urine samples were tested in one of the mentioned laboratories. Those with nonsignificant bacteriuria were excluded. Significant bacteriuria was defined as isolation of a single organism in quantitative counts ≥10^5^ colony-forming units (CFU)/mL from a clean-catch voided urine specimen in asymptomatic patients [9] and ≥10^2^ colony-forming units (CFU)/mL for symptomatic patients [10]. Information regarding age, sex, past medical history, history of exposure to antibiotics within the past 3 months, history of hospital admission within the past 3 months, pregnancy status, and indication for performing urine culture were recorded prospectively using a checklist. Isolated pathogens from urine samples and their antimicrobial susceptibility profile were also recorded.

All three clinical laboratories received midstream urine samples from each patient in a sterile container. The samples were analyzed within 2 h of collection. For this purpose, 10 *μ*l of each urine sample was inoculated on routine culture media, i.e., blood agar and MacConkey agar, using a standard loop. After an overnight incubation at 35°C, the cultures were inspected for the presence of bacterial growth. Any mixed growth of bacterial colonies suggestive of contamination was excluded from further analysis. Finally, bacterial isolates were identified using conventional biochemical tests, and the Kirby–Bauer disk diffusion method was used for antimicrobial susceptibility testing (AST). Then, the AST results were interpreted according to the breakpoints defined by the Clinical and Laboratory Standards Institute (CLSI) for uropathogens.

### 2.1. Statistical Analysis

Continuous data were described with mean and standard deviation and categorical variables with frequency and percentage. Two-sample *t*-test and Mann–Whitney test were used for continuous variables, whereas Fisher's exact test and chi-square tests were used for categorical variables, as appropriate. Significant predictors among the tested independent variables were analyzed by using the binary logistic regression test.

### 2.2. Ethical Approval

The ethics committee of Mashhad University of Medical Sciences approved this study with the code of IR.MUMS.fm.REC.1395.542.

## 3. Results

From October 2017 to June 2019, 424 cases were included in the study, of whom 330 had significant bacteriuria and were analyzed (Table 1). Urine culture was requested for diagnosis of symptomatic UTIs in 240 (82.2%) of 292 cases and screening of asymptomatic bacteriuria in 52 cases (17.8%).

The median of age was 47 (IQR 30, 62) years, with the age range of 1–88 years. Two hundred seventy-six (83.6%) cases were female, of whom 20 (7.2%) were pregnant. Sixty-two (21.8%) had cardiovascular disorders, 27 (21.8%) had diabetes mellitus, 7 (2.5%) had chronic kidney disease/end-stage renal disease (CKD/ESRD), and 5 (1.8%) received kidney transplant. One hundred ninety-two (65.1%) had a previous history of UTIs, with a median interval of 9 (IQR, 3–24) months, 146 (49.7%) mentioned exposure to antibiotics in the past 3 months, and 32 (10.8%) had history of hospital admission in the past 3 months. Twenty-two (7.5%) cases had urinary tract catheter.

The most common uropathogen among 330 isolates was *Escherichia coli* in 201 (60.9%), followed by *Klebsiella* species in 46 (13.9%), *Enterococci* in 27 (8.2%), coagulase-negative staphylococci in 20 (6.1%), *Staphylococcus aureus* in 10 (3%), *Citrobacter freundii* in 6 (1.8%), *Pseudomonas aeruginosa* in 5 (1.5%), and *Proteus mirabilis* in 4 (1.2%).

Analysis of antimicrobial susceptibility of uropathogenic *E. coli* isolates showed the highest susceptibility to nitrofurantoin (89.3%), followed by cefixime (75%) and gentamicin (72.4%) (Table 2). Among *Klebsiella* species, it was highest to ceftriaxone (86.2%), followed by cefixime (81.8%) and ciprofloxacin (72.4%). All the uropathogenic *Enterococci* were susceptible to nitrofurantoin, and their susceptibility rate to ampicillin was 92.3%.

Univariate analysis showed that exposure to antibiotics in the past 3 months was a significant predictor of resistance to ciprofloxacin among uropathogenic *E. coli* isolates (OR: 2.89, 95% CI: 1.33–6.28;*P* value: 0.007) (Table 3). Other tested variables, including different age groups (i.e., children, young adults, and the elderly), gender, diabetes mellitus, history of previous episodes of UTIs, and history of hospital admission in the past 3 months were not significantly associated with resistance to ciprofloxacin. The only predictor of resistance to TMP-SMX among uropathogenic *E. coli* isolates was older age (OR: 2.06, 95% CI: 1.07–3.97; *P* value: 0.032).

## 4. Discussion

Our study showed that community-acquired uropathogenic *Escherichia coli* isolates were highly resistant to trimethoprim-sulfamethoxazole (TMP-SMX) and ciprofloxacin, with resistance rates of 55% and 40%, respectively. According to the 2011 guideline of the IDSA on uncomplicated cystitis and pyelonephritis, local resistance rates of uropathogens that exceed 20% for TMP-SMX and 10% for fluoroquinolones make these antibiotics less appropriate choices for empirical treatment of uncomplicated UTIs [5]. Wide ranges of resistance rates to different antibiotics among uropathogens have been reported by previous studies on UTIs in Iran. Many of these studies lacked clear case definitions and did not determine whether the isolates were community- or hospital-acquired [13–15], or included a mixed population of community- and hospital-acquired isolates [16–19]. This limitation significantly decreases their practical value due to the different nature of uropathogens in the community and hospital settings [2]. Local resistance rates that are reported based on the antibiograms of hospital-acquired bacteria often reflect resistance rates among the uropathogens that were obtained from inpatients, or those with complicated or recurrent infections. Thus, they probably overestimate the rates of resistance among patients with uncomplicated UTIs [1]. There are, however, several studies that mainly focused on community-acquired uropathogens in Iran and similarly reported high rates of resistance to TMP-SMX (32%–76%) [11, 20–25] and ciprofloxacin (32%–48%) [11, 20, 22, 23] among *E. coli* strains. The high resistance rates among community-acquired uropathogenic *E. coli* strains in our study and most of the previous studies in Iran could be at least partly attributable to the high level of antibiotic consumption in the country, with 38.78 defined daily doses of antibiotic per 1000 people [12]. Recent or current antibiotic use has been reported as an important factor in the emergence of resistance among uropathogens [3, 6, 26]. The resistance rates of less than 10% to ciprofloxacin were reported by two studies from Iran that excluded cases with recent exposure to antibiotics [24, 25]. Exposure to antibiotics in the past 3 months was also identified as a significant predictor of resistance to ciprofloxacin among uropathogenic *E. coli* strains (OR: 2.8) in our study. However, we did not investigate the type of antibiotics received by patients, nor their duration or dose before performing the urine culture, the factors that might affect the resistance pattern of isolated uropathogens.

Nitrofurantoin monohydrate/macrocrystals has been recommended as an appropriate choice for the treatment of acute uncomplicated cystitis due to minimal resistance and propensity for ecologic adverse effects of antimicrobial agents (i.e., selection for colonization or infection with multidrug-resistant organisms—so-called “collateral damage”) [5,27]. It was the most active antimicrobial agent with about 90% susceptibility rate among *E. coli* isolates in our study. Similarly, most of the previous studies in Iran reported a high susceptibility rate of 87–97% to nitrofurantoin among community-acquired uropathogenic strains of *E. coli* [11, 24, and 25]. So, it is an appropriate antibiotic for empiric treatment of community-acquired uncomplicated cystitis in Iran. Cefixime and gentamicin may be considered the second-choice alternatives, with susceptibility rates of 75% and 72%, respectively. Nevertheless, previous studies reported wide ranges of susceptibility rates among community-acquired uropathogenic strains of *E. coli* to these two antibiotics, with 56–80% susceptibility rates to cefixime [11, 25] and 49–97% to gentamicin [11, 23–25]. Thus, conclusions regarding appropriateness of these antibiotics as empirical treatment for community-acquired uncomplicated UTIs in Iran cannot be drawn and it has to be determined in the future large-scale studies. In addition, cefixime and other cephalosporins are not generally considered as an optimal option for treatment of uncomplicated UTIs due to their propensities for collateral damage [5]. Amoxicillin, ampicillin, cephalexin, and tetracycline, on the other hand, were associated with highest resistance rates among uropathogenic strains of *E. coli* and *Klebsiella*, as compared to other classes of antibiotics. These findings make these agents less appropriate antimicrobials for empiric therapy of community-acquired uncomplicated UTIs in the country.

*Enterococci* were the third most common uropathogens that were isolated in our study. Ampicillin and nitrofurantoin are considered among the best options for oral treatment of enterococcal cystitis, if susceptibility is documented [28]. All the uropathogenic *Enterococci* and more than 90% of them in our study were susceptible to nitrofurantoin and ampicillin, respectively. Similarly, high susceptibility rates of more than 95% to both nitrofurantoin and ampicillin were reported among community-acquired uropathogenic *E. faecalis* isolates in the study of Karimi et al. in Tehran, Iran [29]. Thus, these two antibiotics can be considered appropriate agents for empiric therapy of community-acquired enterococcal cystitis in Iran.

Our study had some limitations that should be pointed out. Although using a detailed checklist and recording the patients' information prospectively were methodological strengths compared to most of the previous studies in the country, we did not investigate the type of antibiotics received by patients, nor their duration or dose before performing the urine culture, the factors that might affect the resistance pattern of isolated uropathogens. Furthermore, a larger sample size of patients is needed for making more accurate estimation of resistance rates to different antimicrobials among uropathogens and to determine the individual-level predictors of resistance.

## 5. Conclusion

*E. coli* and *Klebsiella* species accounted for about two-thirds of community-acquired uropathogens in our study. In regard to the high susceptibility rates, nitrofurantoin was identified as the first-choice agent for empirical treatment of community-acquired uncomplicated cystitis, while cefixime and gentamicin might be second-choice alternatives that can also be appropriate in those with pyelonephritis. Ciprofloxacin and TMP-SMX, on the other hand, cannot be considered appropriate agents for empiric therapy of UTIs, particularly in those who had exposure to antibiotics in the past 3 months and the elderly.

## Figures and Tables

**Table 1:** Characteristics of patients.

Age (years)	40 (30, 62)
Age groups	
Children (1 mon–<15 yrs)	27 (8.2)
Young adults (≥15–<65 yrs)	240 (72.9)
The elderly (≥65 yrs)	62 (18.8)
Gender (female, %)	276 (83.6)
Underlying comorbidities	
Cardiovascular disorders	62 (21.8)
Diabetes mellitus	57 (20.1)
CKD/ESRD	7 (2.5)
Kidney transplant	5 (1.8)
Others	17 (5.9)
Patients with no underlying comorbidities	156 (54.9)
Urinary catheter	22 (7.5)
Pregnant	20 (6.7)
Exposure to antibiotics within the past 3 months	146 (49.7)
Hospital admission within the past 3 months	32 (10.8)
Previous history of UTIs	192 (65.1)
Isolated uropathogens	
*Escherichia coli*	201 (60.9)
*Klebsiella* species	46 (13.9)
*Enterococci*	27 (8.2)
CoNS	20 (6.1)
*Staphylococcus aureus*	10 (3)
*Citrobacter freundii*	6 (1.8)
*Pseudomonas aeruginosa*	5 (1.5)
*Proteus mirabilis*	4 (1.2)
Others	11 (3.3)

CKD: chronic kidney disease; ESRD: end-stage renal disease; CoNS: coagulase-negative staphylococci. Values are represented by the number of patients with specific characteristics (percent) for nominal variables and by median (25^th^ quartile, 75^th^ quartile) for quantitative variables. None of the patients was hospitalized at the time or a few days before they were included in the study.

**Table 2:** Antimicrobial susceptibility profile of the most common isolated uropathogens.

	*E. coli*	*K. pneumoniae*	*Enterococci*
Ampicillin	2/34 (5.9)	0/10 (0)	12/13 (92.3)
Amoxicillin	8/42 (11)	0/12 (0)	2/3 (66.7)
Nalidixic acid	54/123 (43.9)	16/25 (64)	NA
Ciprofloxacin	71/120 (59.2)	21/29 (72.4)	NA
Gentamicin	113/156 (72.4)	25/36 (69.4)	3/5 (60)
Ceftriaxone	67/109 (61.5)	25/29 (86.2)	NA
Cefixime	27/36 (75)	9/11 (81.8)	NA
Cephalexin	1/26 (3.8)	4/9 (44.4)	NA
Tetracycline	5/40 (12.5)	5/13 (38.5)	8/24 (33.3)
Nitrofurantoin	108/121 (89.3)	18/30 (60)	12/12 (100)
TMP-SMX	69/153 (45.1)	21/34 (61.8)	NA

NA: not applicable; TMP-SMX: trimethoprim-sulfamethoxazole.

**Table 3:** Univariate analysis of the risk factor for community-acquired urinary tract infections caused by ciprofloxacin-resistant *E. coli* strains.

Characteristics	Ciprofloxacin		*B*	OR (95% CI)	*P* value	TMP-SMX		*B*	OR (95% CI)	*P* value
		Resistant				Sensitive	Resistant				Sensitive
Age group (years)	0–14	2 [12]	6 (75)	0.081	1.084 (0.496–2.370)	0.839	4 (33.3)	8 (66.7)	0.721	2.057 (1.066–3.972)	0.032
15–64	40 (43)	53 (57)	46 (41.4)				65 (58.6)				
≥65	7 (36.8)	12 (63.2)	19 (63.3)				11 (36.7)				

Gender (female)		40 (40)	59 (59.6)	0.101	1.106 (0.427–2.869)	0.835	61 (48)	66 (52)	−0.732	0.481 (0.195–1.186)	0.112

Diabetes mellitus		14 (58.3)	10 (41.7)	0.432	1.540 (0.473–5.011)	0.473	12 (46.2)	14 (53.8)	−0.049	0.952 (0.350–2.589)	0.924

History of previous episodes of UTIs		35 (46.7)	40 (53.3)	0.714	2.042 (0.904–4.609)	0.068	46 (54.1)	56 (54.9)			

Exposure to antibiotics in the past 3 months		30 (54.5)	25 (45.5)	1.063	2.894 (1.333–6.285)	0.007	34 (44.2)	43 (55.8)	0.087	1.091 (0.566–2.102)	0.796

History of hospital admission in the past 3 months		9 (64.3)	5 (35.7)	1.077	2.937 (0.916–9.419)	0.070	8 (50)	8 (50)	0.292	1.339 (0.474–3.787)	0.582

TMP-SMX: trimethoprim-sulfamethoxazole.

## Data Availability

The data used to support the findings of this study are included within the article.
